# Genomic variation in microbial populations inhabiting the marine subseafloor at deep-sea hydrothermal vents

**DOI:** 10.1038/s41467-017-01228-6

**Published:** 2017-10-24

**Authors:** Rika E. Anderson, Julie Reveillaud, Emily Reddington, Tom O. Delmont, A. Murat Eren, Jill M. McDermott, Jeff S. Seewald, Julie A. Huber

**Affiliations:** 1 Josephine Bay Paul Center, Marine Biological Laboratory, Woods Hole, MA 02543 USA; 2 Department of Biology, Carleton College, Northfield, MN 55057 USA; 3 Department of Medicine, University of Chicago, Chicago, IL 60637 USA; 4 Marine Chemistry and Geochemistry, Woods Hole Oceanographic Institution, Woods Hole, MA 02543 USA; 5 Cirad UMR 117, Inra UMR 1309 ASTRE, Cirad Campus International de Baillarguet, Montpellier, France; 6 Great Pond Foundation, Edgartown, MA 02539 USA; 7 Department of Earth and Environmental Sciences, Lehigh University, Bethlehem, PA 18015 USA

## Abstract

Little is known about evolutionary drivers of microbial populations in the warm subseafloor of deep-sea hydrothermal vents. Here we reconstruct 73 metagenome-assembled genomes (MAGs) from two geochemically distinct vent fields in the Mid-Cayman Rise to investigate patterns of genomic variation within subseafloor populations. Low-abundance populations with high intra-population diversity coexist alongside high-abundance populations with low genomic diversity, with taxonomic differences in patterns of genomic variation between the mafic Piccard and ultramafic Von Damm vent fields. Populations from Piccard are significantly enriched in nonsynonymous mutations, suggesting stronger purifying selection in Von Damm relative to Piccard. Comparison of nine *Sulfurovum* MAGs reveals two high-coverage, low-diversity MAGs from Piccard enriched in unique genes related to the cellular membrane, suggesting these populations were subject to distinct evolutionary pressures that may correlate with genes related to nutrient uptake, biofilm formation, or viral invasion. These results are consistent with distinct evolutionary histories between geochemically different vent fields, with implications for understanding evolutionary processes in subseafloor microbial populations.

## Abstract (web summary)

The warm subseafloor at deep-sea hydrothermal vents hosts diverse microbial communities. Here, Anderson et al. reconstruct 73 metagenome-assembled genomes from two geochemically distinct hydrothermal vent fields, showing different patterns of genomic variation among diverse microbial taxa.

## Introduction

Marine exploration over the last 40 years has resulted in remarkable discoveries regarding the extent, diversity, and function of life in the deep ocean. Studies focused on chemosynthetic ecosystems at hydrothermal vents^1, 2^ and active microbes buried in sediments kilometers beneath the seafloor^3, 4^ have greatly enhanced our understanding of the intimate connections between the biosphere and geosphere. Despite this advancing knowledge about life in the deep ocean, our understanding of microorganisms in the rocky oceanic crust and the fluids flowing through it remains limited due to the difficulties of accessing these subseafloor habitats.

Low-temperature hydrothermal fluids venting at the seafloor provide access to subseafloor environments and can be analyzed to infer microbial and geochemical processes occurring in the oceanic crust^5–8^. Geochemical environments within hydrothermal systems range from mafic systems with reduced, low pH, metal-, and sulfide-enriched fluids to ultramafic systems with high pH, hydrogen-, and organic-enriched fluids^9^. The mixing of hydrothermal fluids with seawater in subseafloor environments creates physiochemical gradients that support diverse microbial communities. Marker gene surveys have revealed distinct population structures both within^10–13^ and between^14–16^ vent fields that are consistent with variations in fluid geochemistry. However, we have limited knowledge of the evolutionary processes influencing microbial communities in the marine subsurface. Examination of genomic variation within microbial populations can provide insights into the evolutionary and ecological factors that drive diversification, but few studies have focused on genomic variation of microbial populations in the deep sea, despite the fact that the subseafloor is a unique environment for the study of evolution due to its extreme habitats, variations in dispersal rates, metabolic innovation, and novel taxa^17, 18^. Recent work examining genomic variation in microbes inhabiting subseafloor sediments revealed no evidence for changing rates of genome diversification or selection with depth^19^, but studies investigating patterns of selection and variation in microbial populations have not been carried out within and across hydrothermal environments.

Previous work suggests that the genomes of hydrothermal vent microorganisms are extensively variable. Hydrothermal vent metagenomes are enriched in mobile genetic elements, which may represent a means of adapting to the dynamic, gradient-driven vent environment^20, 21^. Comparative genomic studies of *Thiomicrospira* from Lost City^22^, *Pyrococcus furiosus* from Vulcano Island^23^, and *Lebetimonas* from fluid venting at an actively erupting deep-sea volcano^24^ reveal extensive genome rearrangement, gene gain and loss, evidence of horizontal gene transfer, and abundant genomic islands. While individual studies have focused on genome variation within a few specific taxa within vent settings, we do not know whether specific taxa exhibit more strain-level variation than others in the vent environment, or the extent to which divergent strains are partitioned across niches according to their genomic features.

The Mid-Cayman Rise, an ultraslow spreading ridge in the Caribbean Sea, offers an ideal natural laboratory in which to investigate genomic variation in microbial populations inhabiting distinct subseafloor habitats. The Mid-Cayman Rise hosts two geologically distinct vent fields: Piccard, a 4950 m deep mafic-hosted vent field that is the deepest vent field discovered to date^25–27^, and Von Damm, an ultramafic-hosted site that is located about 20 km from Piccard on a nearby massif, at a depth of about 2350 m^28, 29^. Previous geochemical studies have shown that end-member hydrothermal fluids at Piccard are acidic and enriched in dissolved sulfide and hydrogen^27, 30^. In contrast, end member hydrothermal fluids at Von Damm are less acidic, contain lower levels of dissolved sulfide, and have substantially higher concentrations of dissolved hydrocarbons relative to Piccard^27, 28, 30^. These geochemical differences appear to strongly influence microbial community structure. Microbial communities in Von Damm diffuse fluids have greater richness and more metabolic diversity than those found at Piccard, with very little overlap in the phylotypes present at each system^16^. Thus, the proximally located Piccard and Von Damm vent fields present a unique opportunity to examine population dynamics and pangenomic variation across both distant and closely related microbial lineages in two geochemically distinct hydrothermal systems.

Here, we use metagenomic sequencing and binning strategies to recover highly complete metagenome-assembled genomes (MAGs) and examine genomic variation within subseafloor microbial populations. By examining of patterns of fine-scale genomic variation within and between populations, we show that the evolutionary histories of microbial populations from different taxa differed between two distinct geochemically distinct hydrothermal vent fields.

## Results

### General characterization of fluid samples and metagenomic data sets

We collected four diffuse flow hydrothermal fluid samples from Piccard in 2012, and from Von Damm we collected five samples in 2012 and six in 2013 (Table 1). The sample temperatures ranged from ~18 °C to 140 °C, with magnesium concentrations ranging from 14–53 mmol/kg, reflecting the high variability in mixing of seawater with hydrothermal fluid in the subseafloor. Fluid pH ranged from 5.9 to 7.6 (Table 1).

**Table 1:** Information about samples collected from each venting site

**Sample name**	**Year**	**Vent field**	**Vent name**	**T (°C)**	**Cells/ml**	**pH**	**Mg (mmol/kg)**	**# reads in meta-genome**	**Shannon–Wiener Index (16S rRNA mapping)**	**Shannon–Wiener Index (bins)**
FS841	2012	Von Damm	Old Man Tree	114	NC	5.89	14	53,842,644	189.83	NA
FS842	2012	Von Damm	Ravelin #2	86	NC	6.26	15.8	64,300,743	175.35	0.64
FS844	2012	Von Damm	Shrimp Hole	50	2.69E + 05	7.55	47.1	103,787,346	145.76	2.05
FS848	2012	Von Damm	Ginger Castle	47	1.08E + 05	6.65	37.3	77,333,359	105.03	0.693
FS849	2012	Von Damm	Main Orifice	109	2.97E + 04	7.13	47.4	26,061,468	186.84	NA
FS851	2012	Piccard	Hot Chimlet, BVM	106	5.45E + 04	6.98	52.2	75,912,423	140.51	NA
FS852	2012	Piccard	Shrimp Canyon, BVM	44	5.20E + 04	6.45	49.1	37,753,638	63.08	NA
FS854	2012	Piccard	Marker X-19 at BV #4, BVM	18	7.27E + 04	6.79	51.2	140,664,299	91.66	1.97
FS856	2012	Piccard	Shrimp Gulley #2, BSM	108	1.40E + 05	6.66	50.9	104,123,722	78.42	1.79
FS866	2013	Von Damm	Near Main Orifice	130	3.72E + 05	6.01	27.3	24,088,594	168.83	1.73
FS872	2013	Von Damm	Shrimp Hole	30	1.54E + 05	7.61	53.0	40,397,883	159.81	NA
FS874	2013	Von Damm	Twin Peaks	140	4.02E + 05	6.00	15.5	65,834,175	45.36	1.10
FS877	2013	Von Damm	Shrimp Buttery	131	1.81E + 05	6.22	27.3	152,063,932	107.02	1.10
FS879	2013	Von Damm	Hot Cracks #2	29	1.12E + 05	6.91	48.0	24,723,930	80.57	1.04
FS881	2013	Von Damm	Old Man Tree	114	9.41E + 04	6.01	19.0	167,339,253	163.11	1.93

BSM: Beebe Sea mound, BVM: Beebe Vents mound, BWM: Beebe Woods mound, NC: not counted, NA: not applicable

Metagenomic library preparation and high-throughput sequencing resulted in 25–178 million high-quality paired-end reads for each sample (Supplementary Data 1). Phylogenetic analysis of metagenomic reads mapping to 16S ribosomal RNA (rRNA) genes indicated that most samples were dominated by Epsilonproteobacteria, with *Sulfurovum* appearing as the most abundant taxon in this group among almost all samples (Supplementary Fig. 1). The community composition of these samples indicated that Von Damm had higher overall diversity than Piccard (average Shannon–Wiener Index for each sample of 138.9 and 93.4, respectively) (Supplementary Fig. 1).

### Binning of metagenomic contigs

We reconstructed a total of 73 vent-specific MAGs manually refined and quality-controlled from 12 metagenomes from both Piccard and Von Damm (Supplementary Fig. 2; Supplementary Data 2) with a minimum completeness of 70% and maximum redundancy of 10%. The varying number of MAGs we recovered from each sample (Supplementary Fig. 2) was most likely due to different sequencing depths and distinct levels of strain-level diversity among samples, which likely caused differences in metagenome assembly and binning. Nevertheless, we were able to recover highly complete MAGs from 12 different metagenomes spanning 17 archaeal and bacterial phyla. We reconstructed a higher number of MAGs from Von Damm compared to Piccard, and the average diversity of the MAGs per sample was roughly equivalent between vent fields (average Shannon–Wiener Index for Von Damm samples = 1.28, average for Piccard samples = 1.88) (Table 1; Supplementary Fig. 2). We recovered MAGs from eight bacterial and four archaeal phyla in samples from Von Damm. The most commonly identified taxa among Von Damm MAGs included Aquificales, Thermoprotei, and Thiotrichales. We reconstructed MAGs from six bacterial phyla and one archaeal phylum at Piccard, many of which are Epsilonproteobacteria.

**Fig. 1:**
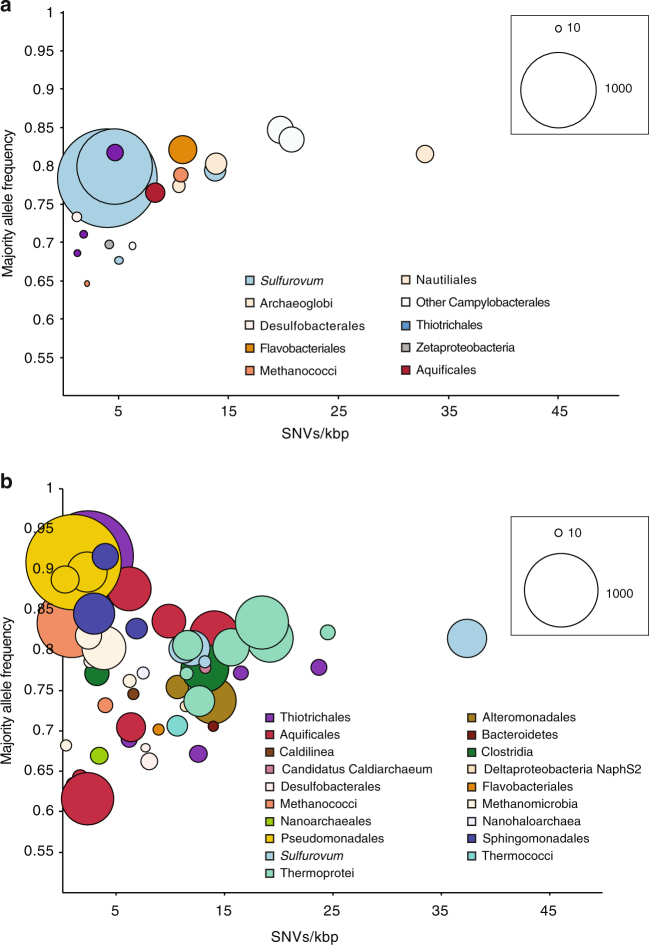
Relationships between SNV density, average majority allele frequency, and mean coverage for MAGs identified from the two vent fields. **a** Piccard vent field, **b** Von Damm vent field. Each bubble represents one MAG. Color of bubble indicates taxonomic assignment. Bubble size indicates average MAG coverage normalized to number of reads in metagenome. Bubble size references represent coverages of 10× and 1000× for a 100 million read metagenome

The average coverage of each MAG varied greatly, ranging from ~10× to over 2000× (Fig. 1; Supplementary Data 2). The MAGs with the highest normalized coverage in Piccard were from the genus *Sulfurovum*. In contrast, the MAGs with the highest coverage at Von Damm included Pseudomonadales, Thiotrichales, Aquificales, Methanomicrobia, and Thermoprotei (Fig. 1; Supplementary Data 2).

### Analysis of genomic variation

We analyzed the nature and extent of genomic variation within the microbial populations represented by each MAG by searching for variants among the reads that mapped to each MAG. The density of single-nucleotide variants (SNVs) varied widely across MAGs (Supplementary Data 2; Fig. 1). We did not observe a direct relationship between SNVs/kbp and coverage, which is consistent with previous studies^31^ and indicates that increased sequencing depth did not bias SNV analyses by including more variant reads (Supplementary Data 2). Different MAGs from the same metagenome with the same taxonomic affiliation may either represent different strains within that taxon, or they may represent different parts of the same “pangenome” of that taxon within the sample.

The MAG-averaged majority allele frequencies, or the percent of reads mapping to a given site that contain the majority SNV, varied widely among MAGs (Fig. 1; Supplementary Data 2; Supplementary Fig. 3). Higher frequencies indicate that a majority allele dominates over minor alleles, whereas frequencies closer to 50% suggest that the alleles occur in fairly even abundance. Although most MAGs had a distribution of allele frequencies around 50–60%, others had a distribution weighted heavily toward a high majority allele frequency, with the bulk of SNVs at ~80% allele frequency (Supplementary Fig. 3). This included a putatively assigned *Thiothrix* MAG from Ravelin #2 and a putative Pseudomonadales MAG from Twin Peaks, both at Von Damm, as well as putative *Sulfurovum* MAGs from Marker X-19 at BV#4 and Shrimp Gully #2, both at Piccard. The vast majority of SNVs we observed were biallelic (only two different bases were observed at that position in the population).

**Fig. 2:**
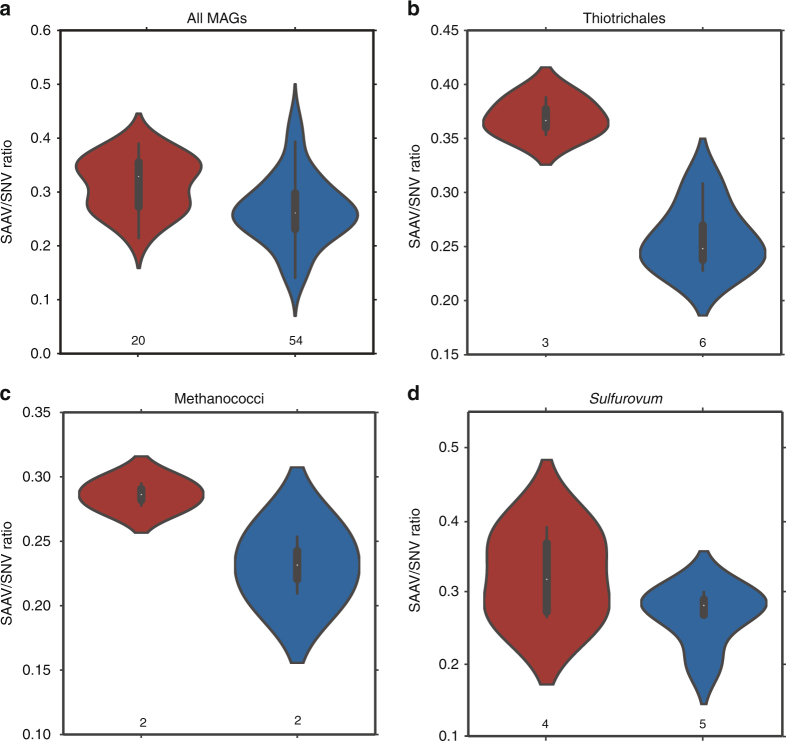
Violin plots showing a kernel density estimation of the underlying distribution of the SAAV to SNV ratio for all MAGs from the Piccard (red) and Von Damm (blue) vent fields. The boxes inside each plot denote the upper and lower quartiles within the distribution, the white dot represents the average. Numbers below each violin indicate the number of MAGs included in that sample. **a** All MAGs from Piccard and Von Damm; **b** Thiotrichales MAGs only; **c** Methanococci MAGs only; **d** *Sulfurovum* MAGs only. Differences in the distribution of the logs of the SAAV/SNV ratio between MAGs from Piccard and Von Damm were significant for all MAGs (*t*-test, *p*: 0.0005) and for Thiotrichales (*t*-test, *p*: 0.0002)

To accurately determine the proportion of SNVs that were not synonymous (i.e., resulting in changes to the amino-acid sequence) in our MAGs, we used a recently introduced strategy for the characterization of “single-amino acid variants” (SAAVs)^32^. Dividing the number of SAAVs by the total number of SNVs in a given MAG offers a similar measure to calculate the ratio of synonymous and nonsynonymous SNPs within a genome (i.e., pN/pS); however, here we counted amino-acid variants only if the read supporting the variant mapped to the entire codon context^32^. We observed that on average, MAGs across various taxa from Piccard had a higher SAAV/SNV ratio than those from Von Damm, and the difference between the logs of the means of the distributions was statistically significant (*t*-test, *p*: 0.0005) (Fig. 2a). We observed a consistent trend within specific taxa for which multiple MAGs from the same taxon were present in both vent fields, though the trend was only significant for Thiotrichales (*p*: 0.0002), which had SAAV/SNV ratios ranging from 0.23 to 0.31 in Von Damm and 0.35–0.39 in Piccard (Fig. 2b). Methanococci showed a similar trend, with SAAV/SNV ratios ranging from 0.21 to 0.25 in Von Damm and 0.28–0.29 in Piccard, but we did not have enough MAGs to determine whether the trend was significant (Fig. 2c). In *Sulfurovum*, the difference in the distributions between the MAGs from the two vent fields was not significant (*t*-test, *p*: 0.18); however, the two MAGs with the highest coverage had the highest SAAV/SNV ratio of all the *Sulfurovum* MAGs (Fig. 2d; Table 2).

**Table 2:** *Sulfurovum* bins identified in all metagenomic samples

**Vent field**	**Vent name**	**Sample**	**Bin number**	**SNVs/kbp**	**Majority allele Freq**.	**SAAVs/SNVs**	**Mean coverage**	**Total length of bin (bp)**	**% Complete**	**% Redundancy**
Von Damm	Shrimp Hole	FS844	Bin_43	11.95	0.8027	0.2999	246.65	1,918,793	97.12	1.44
Von Damm	Shrimp Hole	FS844	Bin_13	13.16	0.7860	0.2009	33.50	1,767,871	92.81	5.76
Von Damm	Old Man Tree	FS881	Bin_43	18.80	0.8005	0.2814	112.98	1,943,705	90.65	2.88
Von Damm	Old Man Tree	FS881	Bin_45	11.19	0.8024	0.2683	273.02	1,736,799	79.86	1.44
Von Damm	Near Main Orifice	FS866	Bin_31	37.36	0.8147	0.2894	71.35	1,835,174	74.82	4.32
Piccard	Mkr X-19 at BV #4, BVM	FS854	Bin_99	4.00	0.7845	0.3898	2441.16	1,870,573	92.09	2.16
Piccard	Shrimp Gulley #2, BSM	FS856	Bin_37	4.66	0.8001	0.3600	1059.15	1,934,456	94.24	4.32
Piccard	Mkr X-19 at BV #4, BVM	FS854	Bin_7	5.06	0.6777	0.2750	18.97	1,625,685	90.65	5.76
Piccard	Mkr X-19 at BV #4, BVM	FS854	Bin_9	13.84	0.7946	0.2651	120.29	1,826,106	75.54	4.32

### Genomic variation within *Sulfurovum* MAGs

**Fig. 3:**
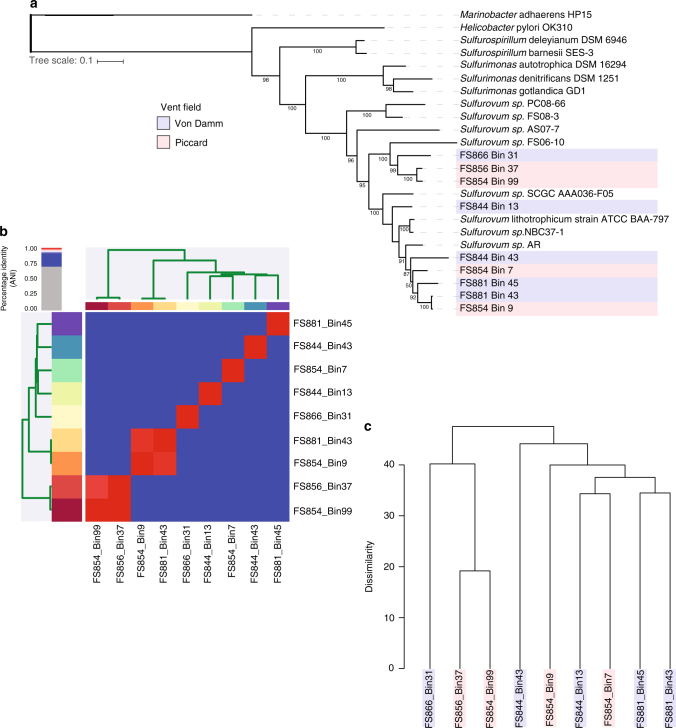
Relationships among *Sulfurovum* MAGs according to universal gene phylogeny, ANI, and gene content. **a** Phylogenetic tree based on concatenated single-copy universal marker proteins. *Sulfurovum* MAGs are indicated in light red (Piccard) and light blue (Von Damm); reference genomes have no color designation. **b** Heatmap and cluster dendrogram grouping MAGs according to ANI. **c** Hierarchical clustering dendrogram based the presence and absence of ORF clusters in MAGs. MAGs are colored according to vent field; red is Piccard, blue is Von Damm

To more closely examine patterns of heterogeneity within a specific and abundant taxonomic group, we focused on five *Sulfurovum* MAGs from Von Damm and four *Sulfurovum* MAGs from Piccard (Table 2). We observed no evidence of biogeographic differentiation among *Sulfurovum* strains based on a phylogenetic tree created from the alignment of 37 single-copy universal marker genes (Fig. 3a). Calculation of average nucleotide identity (ANI) among all nine *Sulfurovum* MAGs indicated that two pairs of MAGs (FS954_Bin99 and FS856_Bin37 from Piccard, and FS854_Bin9 and FS881_Bin43 from Piccard and Von Damm, respectively) were over 98% identical and therefore most likely represented the same species (Fig. 3b). Mapping of metagenomic reads to the MAGs indicated that while each *Sulfurovum* MAG recruited reads primarily from the vent field from which they were recovered, several MAGs recruited reads from both Piccard and Von Damm (Supplementary Fig. 4).

To compare metabolic and functional potential, we searched for the presence and absence of key metabolic genes in all of the *Sulfurovum* MAGs (Supplementary Table 1). Keeping in mind that the absence of key metabolic genes in our MAGs may not necessarily be equivalent to their absence in the actual genome due to the partial recovery of genomes from the environment (completeness of *Sulfurovum* MAGs ranged from 74 to 97%; Table 2), we focused on whether there were large-scale trends in the presence or absence of these key metabolic genes. We included *Sulfurovum lithotrophicum* ATCC BAA-797^33^, *Sulfurovum* sp. AR^34^, and *Sulfurovum* sp. NBC37-1^35^ in the comparison. These three previously sequenced *Sulfurovum* genomes showed some variation in the presence and absence of these key genes, consistent with previous work showing that the genomes of deep-sea Epsilonproteobacteria exhibit a high degree of metabolic plasticity^35^. However, we did not observe clear patterns in the presence or absence of key metabolic genes across vent fields (Supplementary Table 1).

**Fig. 4:**
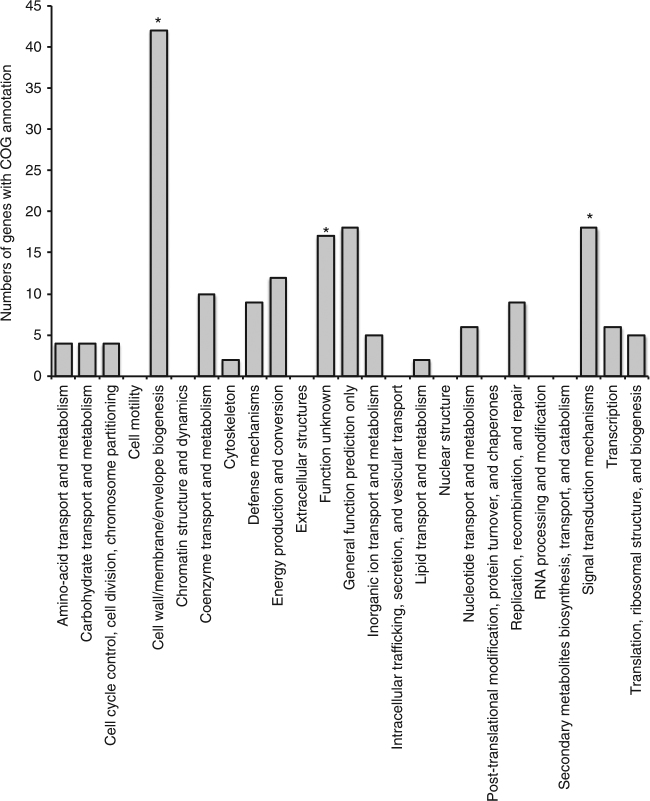
COG categories for all gene clusters that were unique to *Sulfurovum* MAGs. FS854_Bin37 and FS856_Bin99. COG categories that were significantly enriched relative to all genes in all *Sulfurovum* MAGs are indicated with asterisks

Because the key metabolic genes comprised a small percentage of the total genome, we created a cluster dendrogram based on overall gene presence/absence (Fig. 3c). Again, we observed no clear biogeographic distinctions between MAGs from Piccard and Von Damm. However, two MAGs from Piccard exhibited distinct genomic patterns compared to all the others. These two MAGs, FS854_Bin99 and FS856_Bin37, isolated from Shrimp Gulley #2 and X-19 at BV#4 respectively, had higher coverage, lower SNV density, and elevated SAAV/SNV ratios relative to all other *Sulfurovum* MAGs (Supplementary Fig. 5). These two abundant *Sulfurovum* populations were isolated from two different vent sites in Piccard vent field that were located about 50 m apart. One MAG (FS854_Bin99) occurred in high abundance in both X-19 at BV#4 and Shrimp Gulley #2, while the other (FS856_Bin37) was found primarily in Shrimp Gulley #2. These MAGs fell together into the same clade in the phylogenetic tree generated from single-copy universal genes (Fig. 3a) and shared an ANI of 99.9% (Fig. 3b). Moreover, their gene content was more similar to each other than to all other *Sulfurovum* MAGs (Fig. 3c). We were able to identify 310 gene clusters that were shared between these two high-abundance MAGs and absent from all others (Fig. 4; Supplementary Data 3). Among these uniquely shared gene clusters, we observed a statistically significant enrichment of genes that belong to the COG category “cell wall/membrane/envelope biogenesis” (99% confidence interval) as well as “signal transduction mechanisms” (90% confidence interval) and “function unknown” (93% confidence interval) (Fig. 4).

## Discussion

To understand the evolutionary processes occurring within the subseafloor at deep-sea hydrothermal vents, we used MAGs as windows into microbial population diversity and differentiation from two adjacent but geologically distinct deep-sea hydrothermal vent sites. Our analysis of intra-population genomic diversity across the ultramafic-hosted Von Damm and mafic-hosted Piccard vent fields revealed key differences in differential patterns within and between subseafloor microbial populations between the two vent fields.

We observed differences in community membership between Piccard and Von Damm and higher taxonomic diversity in Von Damm vent field, consistent with previous findings showing higher microbial diversity at Von Damm as assessed by 16S rRNA gene amplicon sequencing^16^. These results indicated that there were important differences in community structure between these vent fields. Our examination of coverage, SNV variation, SAAV variation, and allele frequency within each MAG allowed us to determine whether these differences were manifested at the level of population genomic heterogeneity across taxa at Piccard and Von Damm. In both vent fields, we observed variation in SNV density across taxonomically diverse MAGs, which was consistent with other environmental microbial studies that also observed a high degree of variation in genomic heterogeneity across archaeal and bacterial populations^19, 31^.

However, we also observed distinct patterns of population genomic heterogeneity that may reflect distinct evolutionary dynamics operating on different microbial populations. Several MAGs possessed very few SNVs and had high majority allele frequency (corresponding to the upper left corner of Fig. 1). These MAGs generally had high coverage, suggesting that these microbial populations consisted of specific variants that not only grew to high abundance relative to the entire microbial community, but also dominated the population of closely related strains. While they may represent recent migrants into a population, the high abundance of many of these populations is likely more indicative of a clonal expansion. In the Von Damm vent field, these MAGs included Pseudomonadales and Thiotrichales. In contrast, the MAGs showing this pattern in Piccard vent field were putatively assigned as *Sulfurovum*. We hypothesize that the populations represented by these MAGs recently underwent a genome-wide purge of genomic diversity due to a clonal expansion resulting from ecological selection. This observation and interpretation is similar to that observed in natural microbial populations over time in a freshwater lake, where metagenomic binning was also used^31^. MAGs that had a high majority allele frequency and high coverage but slightly higher SNV density (moving toward the upper right portion of Fig. 1), such as several Epsilonproteobacteria MAGs in Piccard and several Thermoprotei or *Sulfurovum* MAGs in Von Damm, most likely represented populations that underwent clonal expansions in the more distant past and had more time to accumulate SNVs in the population. Alternatively, they may represent populations in which the introduction of mutations occurs at a higher rate compared to other populations.

In contrast, several populations displayed markedly different patterns of population genomic heterogeneity. The MAGs in the lower left portion of Fig. 1 had low coverage, a more even diversity of allele variants, and low SNV density, suggesting that these MAGs represent genetically heterogeneous microbial populations that were rare in the community at the time of sampling and were not under strong selection. If conditions were to change such that a specific variant is favored, that variant may then begin to grow in relative abundance and skew the distribution of allele frequencies from more even (~50%) to more uneven (~80–90%). We would expect these MAGs to move from the lower left corner to the upper left corner of Fig. 1, and then accumulate SNVs with time, therefore moving to the upper right corner of Fig. 1. We observed very few MAGs with high SNV density and low majority allele frequency (which would occupy the lower right portion of Fig. 1). However, it is currently unclear whether this reflects true biological trends or whether it is an artifact resulting from the assembly or binning process, in which highly heterogeneous sequences cannot be assembled or clustered into MAGs.

The relative abundance of SAAVs vs. SNVs in a MAG gives an indication of the proportion of variants in a population that affect phenotype. As a result, the SAAV/SNV ratio, like pN/pS, can indicate the strength of selection operating on a population. The significant difference in the overall means of the distribution of SAAVs/SNVs for MAGs recovered from Piccard and Von Damm suggests that, on average, purifying selection is stronger at the Von Damm vent field and/or that positive selection is stronger at Piccard vent field among the populations we observed. We cannot exclude the possibility that non-random recovery of different taxa from each vent field may have biased these results. However, our data showed that this trend was internally consistent within Thiothrichales, and we observed similar trends within other taxa as well. While we cannot identify the precise environmental conditions causing these patterns in the ratio of SAAVs to SNVs, previous work has shown that Von Damm vent field has greater metabolic energy availability and is more geochemically and taxonomically diverse^16, 28^, whereas Piccard vent field is characterized by higher pressure, lower energy availability, and lower geochemical and microbial diversity compared to Von Damm. Thus, we observed distinct patterns of genome heterogeneity within microbial populations from different taxa, which may correspond to the differences in environmental conditions between Piccard and Von Damm.

Previous work has shown that the *Sulfurovum* genus exhibits substantial microdiversity in hydrothermal environments^11, 36^, and it has been hypothesized that this microdiversity results from steep geochemical gradients^37^. Our recovery of multiple *Sulfurovum* MAGs from samples at the Mid-Cayman Rise confirms this microdiversity. Moreover, through analysis of variation at the level of single nucleotides, we were able to show that two specific *Sulfurovum* populations from Piccard were characterized by high coverage and low SNV density ratios compared to all other *Sulfurovum* MAGs, possibly indicating that they were subject to distinct ecological or evolutionary pressures compared to other *Sulfurovum* populations. Moreover, these two MAGs had relatively high SAAV/SNV ratios, indicating a slightly higher abundance of nonsynonymous substitutions relative to other *Sulfurovum* MAGs. Recent work examining SNV-level variation in microbial populations distinguished between old, diverse populations and young, low-diversity populations that recently underwent a selective sweep^38^. Older populations have had time to accumulate SNV mutations and purifying selection has acted to remove nonsynonymous mutations, whereas young, low-diversity populations that have more recently undergone a purge of genomic diversity have fewer SNVs and more nonsynonymous mutations^38^. Previous work shows that these old, diverse populations also tend to maintain their genomic diversity over the course of several years^31, 38^. Thus, the two high-abundance *Sulfurovum* populations at Piccard appear to have undergone clonal expansions or selective sweeps in the more recent past compared to the other *Sulfurovum* populations, which may represent older, more diverse populations that have had time to accumulate genomic variation.

Characterization of conserved single-copy genes as well as their pangenome showed that these two high-abundance, low diversity *Sulfurovum* populations were very similar to each other, suggesting they possessed genomic features in common enabling them to rapidly grow to high abundance in their respective habitats. We identified multiple gene clusters distinguishing these two closely related populations from the other *Sulfurovum* populations. While any of these genes may have provided a selective advantage to the lineages represented by these MAGs, their uniquely shared pangenome was enriched in genes related to cell wall and cell membrane functions, including genes related to membrane proteins, ion channels, and glycosyltransferases. While we cannot definitively state that the presence of these unique genes was responsible for the high abundance of these two *Sulfurovum* populations, the gain or loss of genes related to outer membrane or polysaccharide modification has been previously observed in other microbial lineages, including in vent Epsilonproteobacteria, in which a glycosyltransferase cluster formed part of the variable genome in *Lebetimonas* from NW Rota-1 seamount^24^. Outside of the hydrothermal vent environment, variable genes related to cellular membrane processes have been observed in Haloarchaea^39^, *Salinibacter* ^40^, SAR11^41^, and *Prochlorococcus* ^42^. It is possible that modification, gain, or loss of genes related to the outermost cell surface is subject to strong selection because it acts as the first layer of interaction between a cell and its environment. For example, the removal of outer membrane proteins can deprive viruses of binding sites, potentially allowing the viral host to evade viral infection.

Understanding patterns of natural genomic variation is crucial to understanding how microbial populations evolve. Overall, we show that in two geologically distinct vent fields, low-abundance, high-diversity microbial populations co-existed alongside high-abundance, low-diversity populations that appeared to be under selection or had recently undergone a clonal expansion. We show that the taxa displaying these patterns of population heterogeneity differed between vent fields, suggesting that different taxa were under selection in the mafic-hosted Piccard vent field compared to the ultramafic-hosted Von Damm. In Piccard, two specific *Sulfurovum* populations appeared to have recently undergone a sweep or expansion, and we show that these patterns may be linked to the presence of genes related to outer membrane modification. Thus, through these analyses we can begin to reveal the evolutionary dynamics governing microbial populations inhabiting the subseafloor environment.

## Methods

### Sample collection

We collected diffuse flow hydrothermal fluid samples during cruises aboard the R/V Atlantis in January 2012 (FS841-FS856) and the R/V Falkor in June 2013 (FS866-FS881) (Table 1). The 2012 samples were collected using the Mat sampler^43^ deployed from the ROV Jason, and the 2013 samples were collected with the SUPR version 2 sampler^44^ deployed from the HROV Nereus. We pumped ~3–6 l of fluid through 0.22 µm Sterivex filters (Millipore), flooded the filters with RNALater (Ambion) and sealed them with Luer Caps, stored the filters in sterile Falcon tubes, and froze them at −80 °C. Further details of sample collection and preservation are described in Reveillaud et al.^16^, along with details of chemical analysis for pH and magnesium.

### Microbial community DNA preparation and sequencing

We extracted total genomic DNA from half of the Sterivex filter as described in Akerman et al.^12^ Briefly, 1.85 ml of DNA extraction buffer (0.1 M Tris-HCl, 0.1 M Na_2_-EDTA, 0.1 M NaH_2_PO_4_, 1.5 M NaCl, and 1% cetyltrimethylammonium bromide) was added to the Sterivex filters, followed by DNA extraction as described in Huber et al.^45^ We prepared the metagenomic libraries as described in Reveillaud et al.^16^, using the Ovation Ultralow Library DR multiplex system (Nugen) following manufacturer’s instructions. We sequenced all libraries on an Illumina Hi Seq 1000 at the W.M. Keck Facility in the Josephine Bay Paul Center at the Marine Biological Laboratory. All libraries were sheared at 175 bp using a Covaris S-series sonicator, yielding paired-end reads with a 30 bp overlap. For assembly, we filtered raw reads using the quality filtering technique recommended by Minoche et al.^46^ using the illumina-utils package^47^ v1.4.4 using the program “iu-filter-quality-minoche.” For mapping, we merged and filtered reads also using the illumina-utils package^47^ using the program “iu-merge-pairs” with the flag “–enforce-Q30-check”, and removed sequences with more than two mismatches in the overlapping region with the program “iu-filter-merged-reads.” This resulted in ~170 bp long high-quality reads.

### Taxonomic distribution of metagenomic samples

We determined the relative abundance of different taxa in each metagenome by mapping the reads of each sample to the Silva SSU and LSU Parc databases (release 111)^48, 49^, followed by mapping of matching reads to the Greengenes 13_5 16S rRNA database^50^. We used Bowtie2^51^ v.2.2.9 for mapping using default settings and local alignment. Reads that mapped were classified using mothur^52^ using classify.seqs with the Silva 16S rRNA database, using a cutoff of 50.

### Metagenome assembly, mapping, and binning

We assembled metagenomic reads using idba-ud^53^ v1.1.2 with default settings. We only included contigs of at least 1000 bp in length to ensure robust contig clustering based on tetra-nucleotide frequency and coverage. We mapped the metagenomic reads of each sample to the assembled contigs using bwa aln v0.5.5^54^ with default settings. We used anvi’o v2.1.0^55^ to manually organize the metagenomic contigs of each sample into metagenomic bins based on tetra-nucleotide composition and relative coverage of each contig across all samples. To estimate the completion and redundancy of metagenomic bins, anvi’o used PRODIGAL^56^ v2.6.2 to identify open reading frames in our contigs, and HMMER^57^ v3.1b2 to search for their occurrence in two collections of single-copy core genes for bacteria^58^ and archaea^59^. Using these estimates, we marked 73 metagenomic bins as MAGs using a threshold of <10% redundancy and >70% completion. We did not include MAGs that are known to be native to background deep seawater in our analysis.

We determined the taxonomy of each MAG using PhyloSift^60^, using “phylosift all” with the “–isolate” and “–besthit” flags. We compiled the concatenated protein alignments created by PhyloSift to create maximum likelihood phylogenetic trees using RAxML v.7.2.8^61^ using the “rapid bootstrap” method with 100 bootstraps and the “PROTGAMMAAUTO” model of rate heterogeneity. We designated *Marinobacter adhaerens* HP15 (a Gammaproteobacterium) as the outgroup to root the *Sulfurovum* tree. We visualized the trees using the Interactive Tree of Life website (ITOL) (itol.embl.edu)^62^.

To calculate ANI, we used the Python3 package pyani (https://github.com/widdowquinn/pyani), which calculates ANI between two genomes following the methods described by Richter et al.^63^ We used the “-anib” flag, which uses BLASTN to align 1020 nt fragments of the input FASTA files to calculate ANI among all *Sulfurovum* MAGs.

### Gene calling, annotation, and comparison of MAGs

For functional-based comparison, we used the JGI-IMG^64, 65^ pipeline to call and annotate open reading frames (ORFs) in contigs from each MAG using the KEGG database. We included four reference *Sulfurovum* sequences that were also in the JGI-IMG database for comparison of key metabolic genes (*Sulfurovum* sp. AR, *Sulfurovum* sp. NBC7-1, *Sulfurovum* G1, and *Sulfurovum lithotrophicum* ATCC BA797). For sequence-based comparison, we used the Integrated Toolkit for the Exploration of microbial Pangenomes (ITEP)^66^ to conduct MCL clustering and comparison of ORFs that had been identified using the RAST pipeline^67^. We clustered ORFs using an inflation value of 2 and a maxbit score of 0.3. We used RPSBLAST to compare a representative sequence from each ORF cluster against NCBI’s Conserved Domains Database to obtain the COG annotation for each ORF with a maximum e-value of 1e−05. We used ITEP to generate a presence–absence matrix of all gene clusters among all *Sulfurovum* MAGs. We converted this presence–absence matrix into a dissimilarity matrix and then created a cluster dendrogram using the “hclust” and “plot” functions using default parameters in R 3.3.3^68^. To cluster based on patterns of gene presence/absence among the MAGs, we created a binary gene presence/absence table in ITEP^66^ and then used this to create a distance matrix in R^68^ using the “dist” function with default parameters. We used this distance matrix to create a hierarchical clustering dendrogram in R as described above. We used a non-parametric statistical analysis of the distribution of samples^69^ to determine whether there was a significant enrichment of specific gene types in the uniquely shared genes among *Sulfurovum* MAGs using 500 repetitions and a sample size of 200. We report enrichments within a 90% confidence level and above.

### Single-nucleotide and single-amino acid variant analyses

We used anvi’o 2.1.0 to identify and profile single-nucleotide variants (SNVs) in our MAGs based on mapping of the metagenomic reads from the sample from which the MAG was recovered. Anvi’o relies on a heuristic to identify SNVs that sets a minimum baseline that varies as a function of coverage depth in order to minimize the impact of sequencing or mapping error^55^ (the URL http://merenlab.org/2015/07/20/analyzing-variability/ gives access to a tutorial). We normalized coverage by dividing coverage depth by the total number of reads in the metagenome. We reported allele frequencies based on the frequency of the majority allele. We required all positions to have a minimum coverage of 10 in order to be included in SNV calculations, and only counted positions in which the minimum departure from the consensus (calculated as the total number of reads not matching the consensus divided by the total number of mapped reads) was 0.05. Violin plots of allele frequency were created using the Seaborn Python visualization library based on matplotlib^70^.

We used anvi’o to calculate the number of single-amino acid variants (SAAVs) per MAG using the –engine AA option in the program “anvi-gen-variability-profile,” which reports amino-acid variants that were computed during the profiling of mapping results by the program anvi-profile with the inclusion of the flag --profile-AA-frequencies. For each codon position in open reading frames, the SAAV characterization framework of anvi’o employs only short reads that map to all three codons in a given context, which reduces the impact of noise due partial mappings and spurious inflation of SAAVs by maintaining the physical linkage of bases in a given short read^32^. We used the “car” and “gdata” packages in R 3.3.3 to calculate statistical significance of the difference of means in SAAV/SNV distributions among MAGs in Piccard and Von Damm using the Welch two-sample *t*-test.

We use the term “single-nucleotide variants” (SNVs) rather than the more common term “single nucleotide polymorphisms” (SNPs) because “SNP” is generally used to identify single base pair variants between fully sequenced genomes in the same population, which represent different alleles. Here, we identify variation by mapping metagenomic reads to a metagenomic MAG. While the stringency of mapping used here should ensure that we identify reads from the same population, it is possible that some mapped reads are from more distantly related members of the microbial community that are not part of the same population. We therefore use the term “SNVs” to denote single base pair differences, but operationally they are equivalent to SNPs.

### Data availability

Metagenomic reads are deposited under study accession code PRJEB15541 in the EMBL-EBI European Nucleotide Archive (ENA) database. The anvi’o merged profiles and contigs databases for the metagenomic data and metagenomic MAGs are available on Figshare (figshare.com/projects/Mid-Cayman_Rise_Metagenome_Assembled_Genomes/20783) for re-analysis using anvi’o v2.1.0. The authors declare that all other data supporting the findings of the study are available in this article and its Supplementary Information files, or from the corresponding author upon request.

## Electronic supplementary material

Supplementary Information

Peer Review File

Description of Additional Supplementary Files

Supplementary Data 1

Supplementary Data 2

Supplementary Data 3
