# Supplementary material for: Genomic variation in microbial populations inhabiting the marine subseafloor at deep-sea hydrothermal vents
Source: Nat Commun. 2017 Oct 24;8:1114. doi: 10.1038/s41467-017-01228-6 (PMC5655027; doi:10.1038/s41467-017-01228-6)
Supplement: Supplementary file 1 — Supplementary Information [file 41467_2017_1228_MOESM1_ESM.pdf]

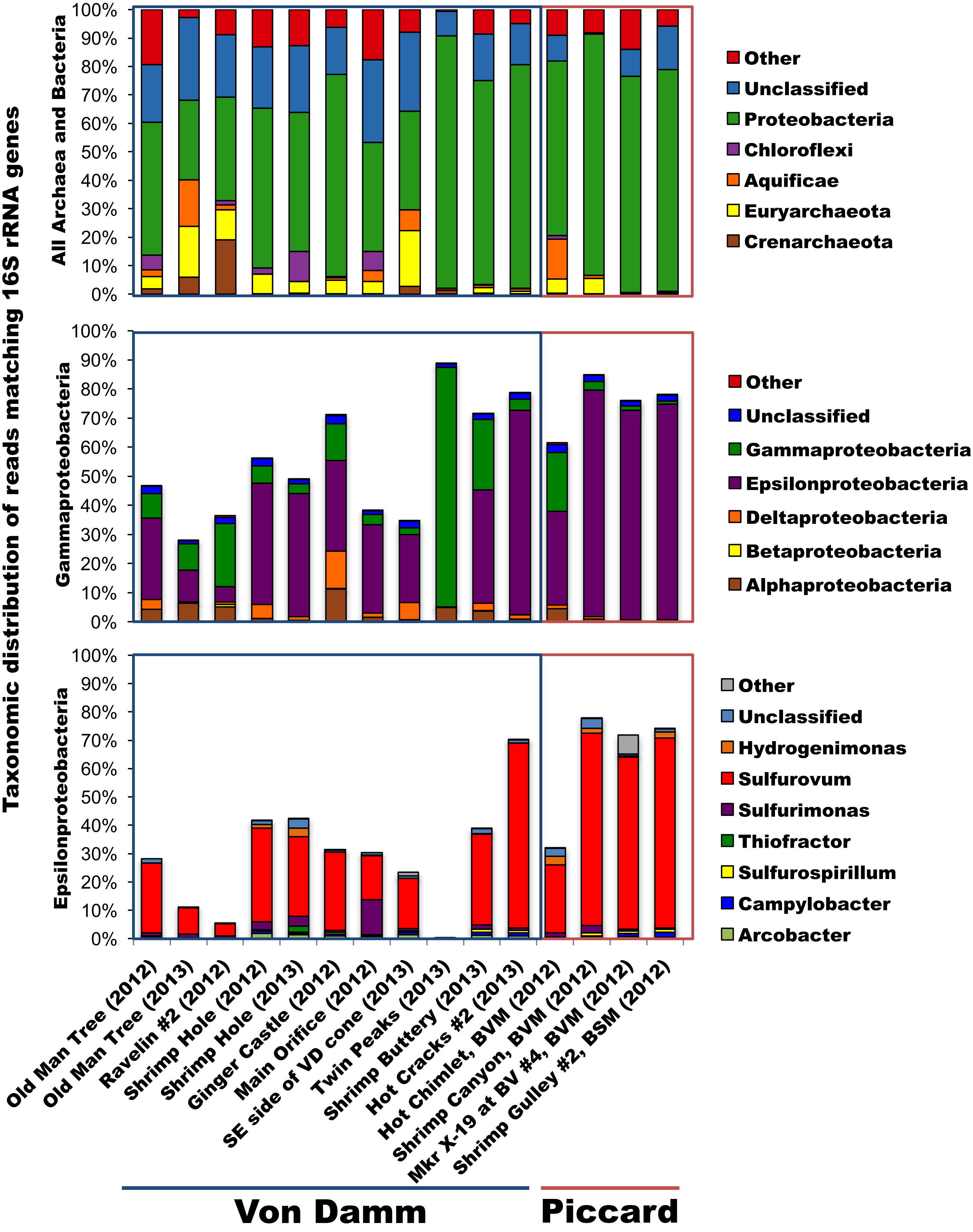

Supplementary Figure 1. Taxonomic distribution of microbial communities in Mid-Cayman Rise samples based on mapping of metagenomic reads to the SILVA 16S rRNA database.

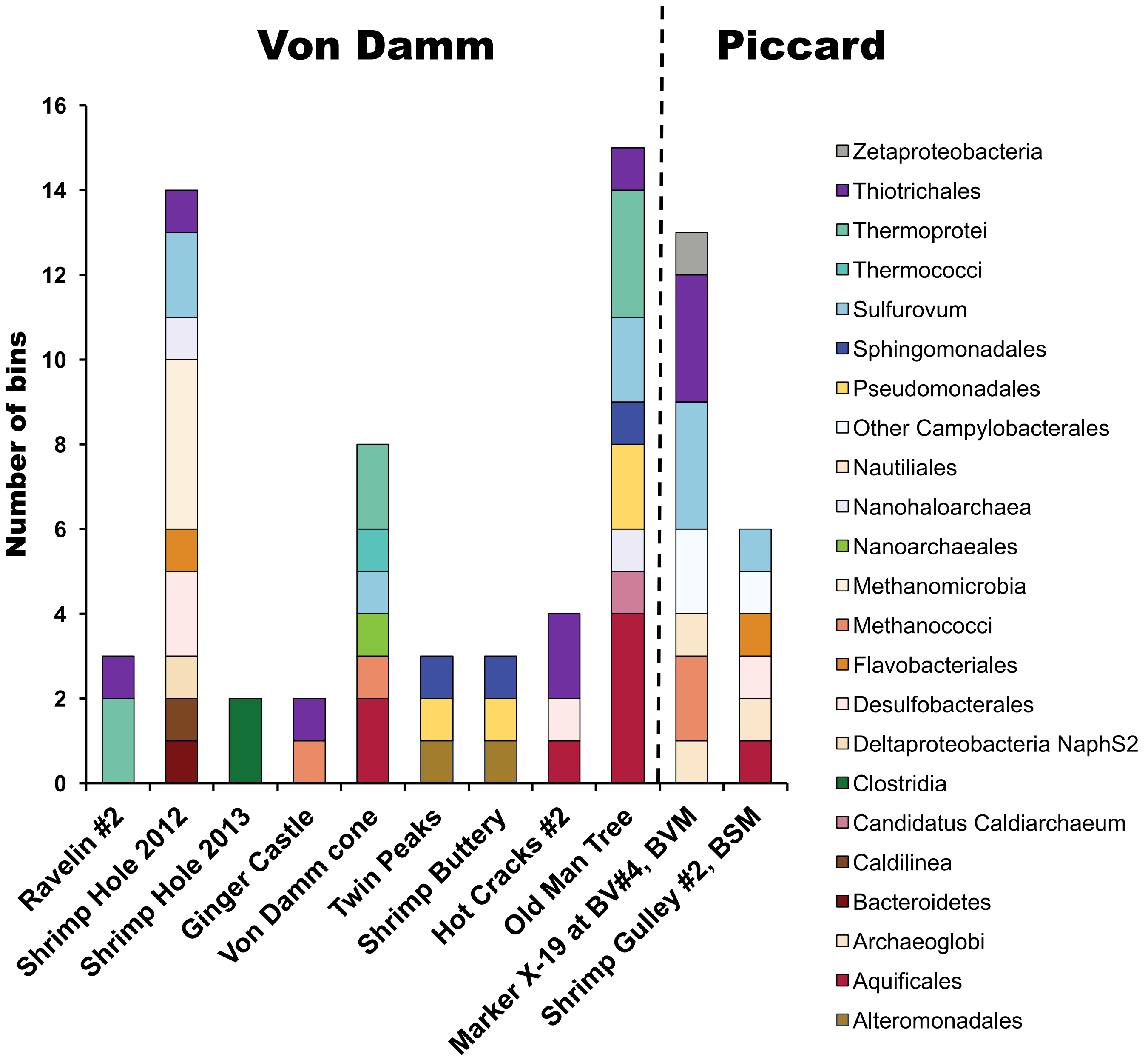

Supplementary Figure 2. Taxonomic category of each MAG identified from all samples on the Mid-Cayman Rise.

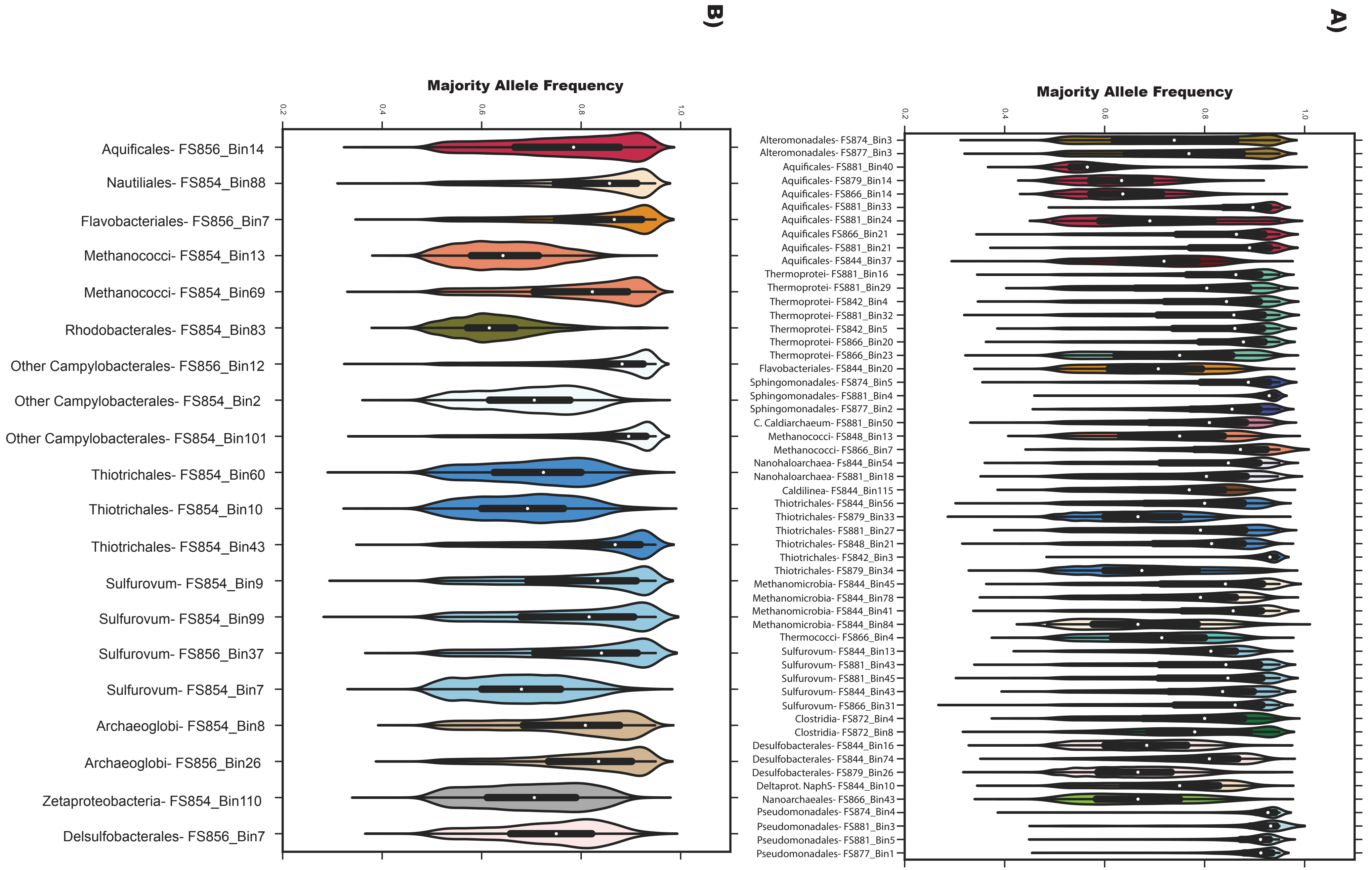

Supplementary Figure 3. Violin plots showing a kernel density estimation of the underlying distribution of majority allele frequencies for all SNVs within all MAGs from the A) Von Damm and B) Piccard vent fields. The boxes inside each plot denote the upper and lower quartiles within the distribution, the white dot represents the average.

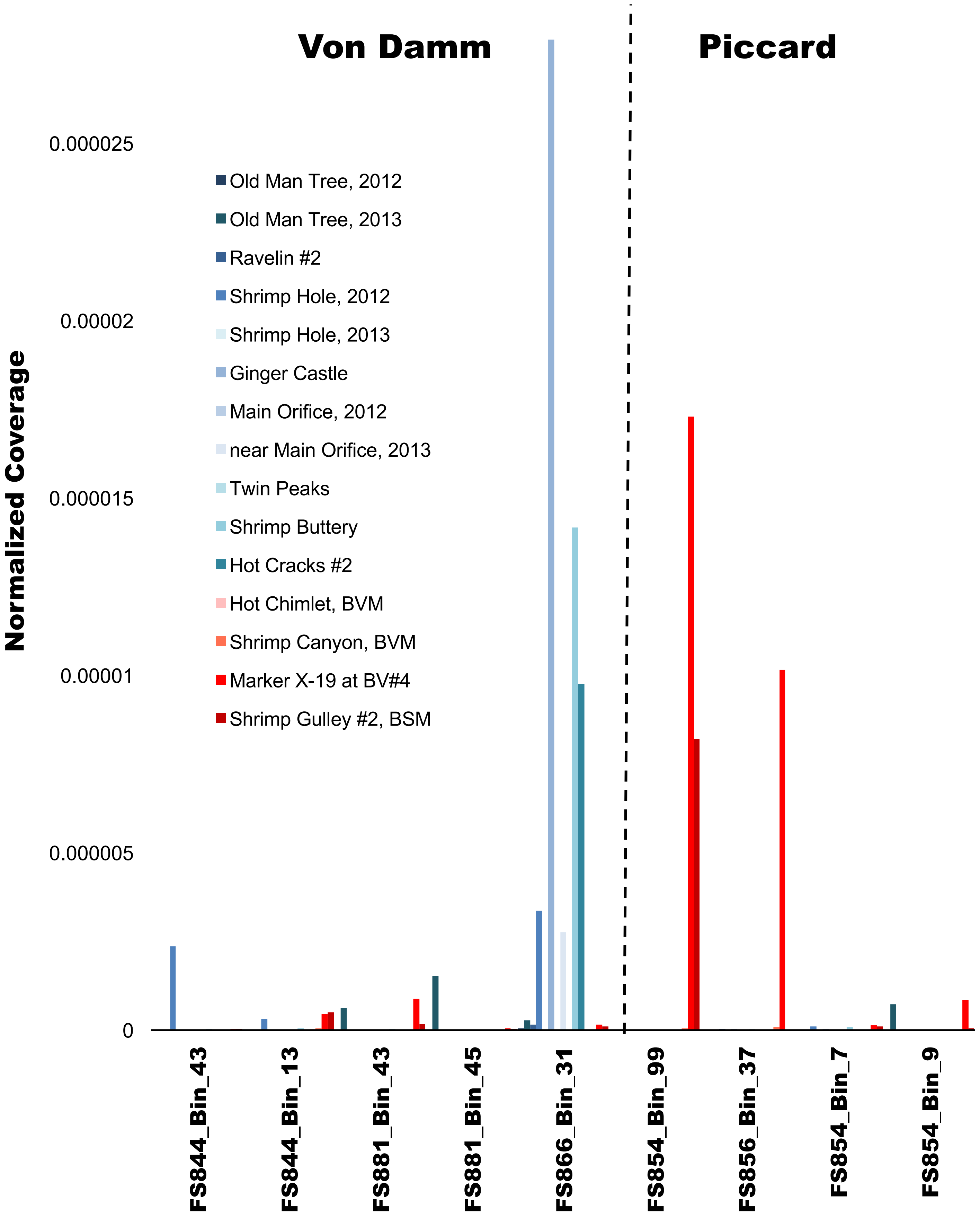

Supplementary Figure 4. Bar chart depicting normalized coverage of all *Sulfurovum* MAGs across samples.

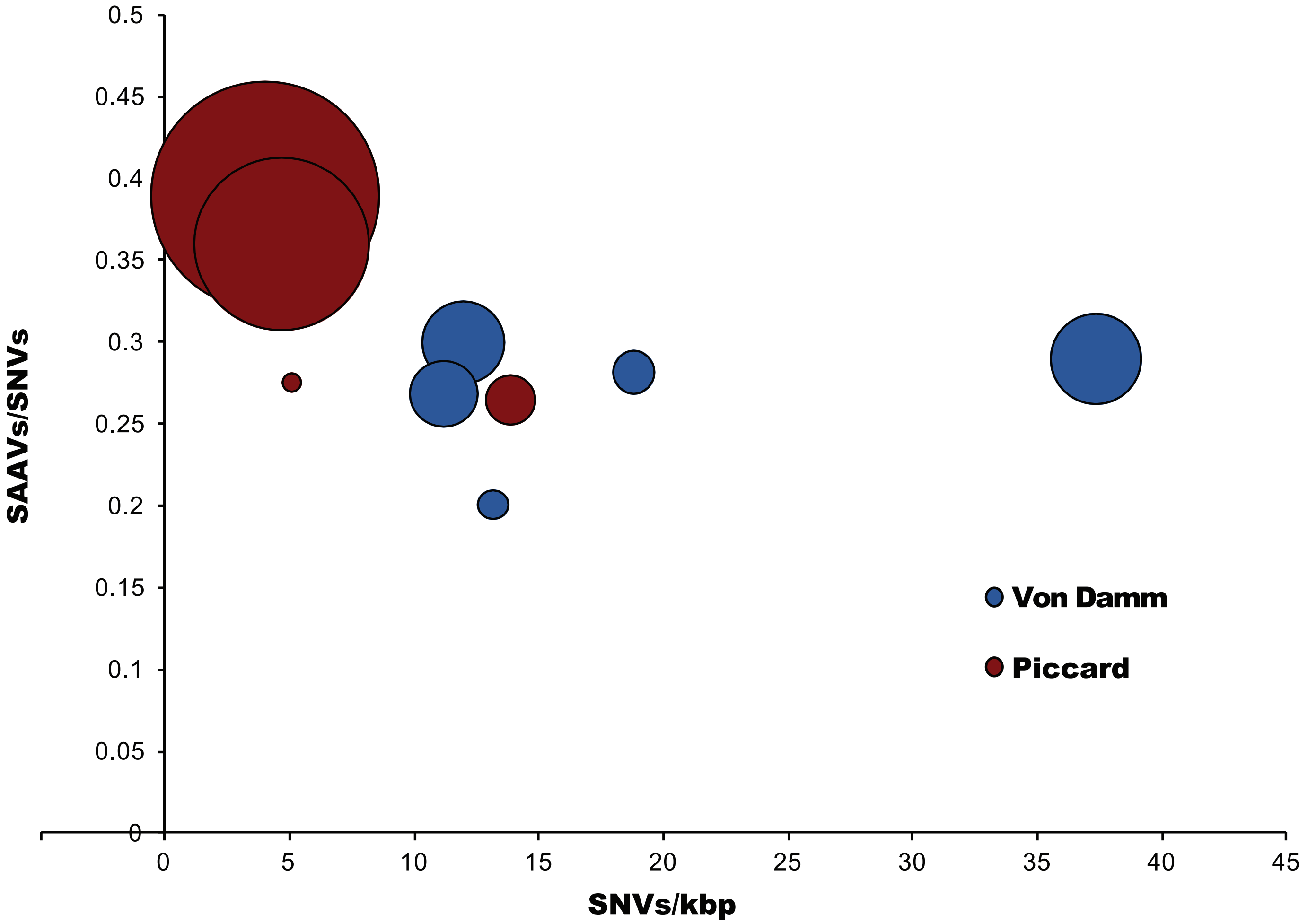

Supplementary Figure 5. Bubble plots showing two *Sulfuoruvm* MAGs with distinctive SNV and AAV patterns compared to other *Sulfurovum* MAGs. SNVs/kbp are on the x-axis, SAAVs/SNVs on the y axis. Size of the bubble represents normalized coverage. FS854_Bin37 and FS856_Bin99 are the two large orange bubbles in the upper left portion of the graph.

Supplementary Table 1. Presence and absence of key metabolic genes in *Sulfurovum* bins. References are in the right-hand columns.

KO Number	Genes	Annotation	Metabolism	Von Damm					Piccard							
				FS844_Bin13	FS844_Bin43	FS866_Bin31	FS881_Bin43	FS881_Bin45	FS854_Bin7	FS854_Bin99	FS854_Bin9	FS856_Bin37	Sulfurovum G1	Sulfurovum sp NBC37−1	Sulfurovum sp. AR	Sulfurovum litothrophicum ATCC BA 797
K00174	korA, oorA, oforA	2-oxoglutarate/2-oxoacid ferredoxin oxidoreductase subunit alpha	carbon fixation	+	+	+		+	+				+	+		+
K00175	korB, oorB, oforB	2-oxoglutarate/2-oxoacid ferredoxin oxidoreductase subunit beta	carbon fixation	+	+	+		+					+	+	+	+
K00176	korD, oorD	2-oxoglutarate ferredoxin oxidoreductase subunit delta	carbon fixation	+	+	+			+				+	+		+
K00177	korC, oorC	2-oxoglutarate ferredoxin oxidoreductase subunit gamma	carbon fixation	+		+							+	+		+
K00239	sdhA, frdA	succinate dehydrogenase / fumarate reductase, flavoprotein subunit	carbon fixation	+	+	+	+	+	+	+	+	+	+	+	+	+
K00240	sdhB, frdB	succinate dehydrogenase / fumarate reductase, iron-sulfur subunit	carbon fixation	+	+	+	+	+	+	+	+	+	+	+	+	+
K00244	frdA	fumarate reductase flavoprotein subunit	carbon fixation						+	+	+			+		+
K00245	frdB	fumarate reductase iron-sulfur subunit	carbon fixation	+		+	+	+	+	+	+	+	+	+	+	+
K15230	aclA	ATP-citrate lyase alpha-subunit	carbon fixation		+		+	+	+				+	+	+	+
K15231	aclB	ATP-citrate lyase beta-subunit	carbon fixation		+		+	+	+	+		+	+	+	+	+
K00368	nirK	nitrite reductase (NO-forming) [EC:1.7.2.1]	denitrification		+											
K00376	nosZ	nitrous-oxide reductase [EC:1.7.2.4]	denitrification		+				+	+		+		+		+
K04561	norB	nitric oxide reductase subunit B [EC:1.7.2.5]	denitrification	+	+		+		+		+			+		+
K15864	nirS	nitrite reductase (NO-forming) / hydroxylamine reductase [EC:1.7.2.1 1.7.99.1]	denitrification	+						+		+				
K02567	napA	periplasmic nitrate reductase NapA [EC:1.7.99.4]	denitrification	+	+	+	+			+	+	+		+		+
K02588	nifH	nitrogenase iron protein NifH [EC:1.18.6.1]	nitrogen fixation										+			
K03620	hyaC	Ni/Fe-hydrogenase 1 B-type cytochrome subunit	hydrogen				+	+		+			+	+	+	
K05922	hydB3	quinone-reactive Ni/Fe-hydrogenase large subunit [EC:1.12.5.1]	hydrogen				+	+	+		+		+	+	+	
K05927	hydA3	quinone-reactive Ni/Fe-hydrogenase small subunit [EC:1.12.5.1]	hydrogen				+	+	+		+		+	+	+	
K06281	hyaB, hybC	hydrogenase large subunit [EC:1.12.99.6]	hydrogen			+	+				+		+			
K06282	hyaA, hybO	hydrogenase small subunit [EC:1.12.99.6]	hydrogen			+	+		+		+		+	+		
K00404	ccoN	cytochrome c oxidase cbb3-type subunit I [EC:1.9.3.1]	O_2 respiration		+	+		+	+		+		+	+	+	+
K00405	ccoO	cytochrome c oxidase cbb3-type subunit II	O_2 respiration	+	+	+	+	+	+		+		+	+	+	+
K00406	ccoP	cytochrome c oxidase cbb3-type subunit III	O_2 respiration	+		+	+	+	+		+		+	+	+	+
K00407	ccoQ	cytochrome c oxidase cbb3-type subunit IV	O_2 respiration	+		+	+	+	+		+		+	+	+	+
K02274	coxA	cytochrome c oxidase subunit I [EC:1.9.3.1]	O_2 respiration				+	+			+			+	+	
K02275	coxB	cytochrome c oxidase subunit II [EC:1.9.3.1]	O_2 respiration				+	+			+			+	+	
K02276	coxC	cytochrome c oxidase subunit III [EC:1.9.3.1]	O_2 respiration				+	+			+				+	
K17218	sqr	sulfide:quinone oxidoreductase [EC:1.8.5.-]	sulfur oxidation	+	+	+	+	+	+	+	+	+	+	+	+	+
K17222	soxA	sulfur-oxidizing protein SoxA	sulfur oxidation		+	+	+			+	+	+		+	+	+
K17223	soxX	sulfur-oxidizing protein SoxX	sulfur oxidation		+	+	+			+	+	+		+		+
K17224	soxB	sulfur-oxidizing protein SoxB	sulfur oxidation		+	+	+			+	+	+		+	+	+
K17225	soxC	sulfane dehydrogenase subunit soxC	sulfur oxidation	+	+	+	+	+	+	+	+	+		+	+	+
K17226	soxY	sulfur-oxidizing protein SoxY	sulfur oxidation	+	+	+	+	+		+	+	+		+	+	+
K17227	soxX	sulfur-oxidizing protein SoxZ	sulfur oxidation	+	+	+	+	+		+	+	+		+	+	+
K17229	fccB	sulfide dehydrogenase [flavocytochrome c] flavoprotein chain [EC:1.8.2.3]	sulfur oxidation	+												
K00958	sat, met3	sulfate adenylyltransferase [EC:2.7.7.4]	sulfur redox	+	+	+	+	+	+	+	+	+	+	+	+	+
