# Supplementary material for: Genomic variation in microbial populations inhabiting the marine subseafloor at deep-sea hydrothermal vents
Source: Nat Commun. 2017 Oct 24;8:1114. doi: 10.1038/s41467-017-01228-6 (PMC5655027; doi:10.1038/s41467-017-01228-6)
Supplement: Supplementary file 3 — Description of Additional Supplementary Files [file 41467_2017_1228_MOESM3_ESM.pdf]

Description of Additional Supplementary Files

File Name: Supplementary Data 1
Description: Data regarding quality filtering and assembly of metagenomic reads.

File Name: Supplementary Data 2
Description: MAGs identified in individual metagenome assemblies from Mid-Cayman Rise samples.

File Name: Supplementary Data 3
Description: COG annotations of all genes that were found in gene clusters that were uniquely shared between two high-abundance Sulfurovum MAGs. We only show genes that had a match to the Conserved Domain Database. This table shows only the top match for each protein to the COG database.
