# Supplementary material for: Genomic variation in microbial populations inhabiting the marine subseafloor at deep-sea hydrothermal vents
Source: Nat Commun. 2017 Oct 24;8:1114. doi: 10.1038/s41467-017-01228-6 (PMC5655027; doi:10.1038/s41467-017-01228-6)
Supplement: Supplementary file 2 — Peer Review File [file 41467_2017_1228_MOESM2_ESM.pdf]

Editorial Note: This manuscript has been previously reviewed at another journal that is not operating a transparent peer review scheme. The manuscript was considered suitable for publication without further review at *Nature Communications.*
